# Nervousness

**Published:** 1860-04-01

**Authors:** 


### Art. VII.—NERVOUSNESS.

Nervousness is one of those *quasi*-technical terms which are to be reprobated for their vagueness, but which cannot be got rid of. We refuse to admit them into strictly scientific nomenclature, but we are not able to ignore their existence. Nervousness is one of the most obnoxious as well as one of the most commonly used of these terms. It is most obnoxious, because in its pathological signification—the *nervousness* of "medical cant" as Johnson would have contemptuously characterized the word—it has a meaning the very antithesis of the one that legitimately belongs to it. Nervousness, the synonym of strength and vigour, and nervousness, the synonym of feebleness and nervelessness, clash together most disagreeably; but although we may carp at this, the word with its latter meaning has an established place. It is used popularly to convey a notion of the many functional deviations, psychical, sensory, and motor, which mark the *nervous diathesis*, and which do not possess a sufficiently distinct character to enable us to classify them with precision under one or other clearly defined form of disease. It serves also to express similar lesions when they occur consecutive to many acute and chronic affections. It is, indeed, a very general term proper to many symptoms arising from widely different sources.

In systematic medicine we do not hesitate to make use of the parent-word *nervous* in its simplest signification, to wit, "relating to the nerves," but the phenomena included commonly under the term nervousness are scattered abroad in various groups, under the heads of the different pathological conditions in which they appear to originate or to be most immediately connected with.

The fitness of this arrangement has been recently questioned by Dr. L. Bouchut.* He holds that the various symptoms for which we have no other term of sufficiently wide scope than

* *De l'Etat Nerveux aigu et chronique, ou Nervosisme.* Par L. Bouchut, Professor agrégé à la Faculté de Médecine de Paris. Paris. 1860.

nervousness, in its popular acceptation, as well as sundry others significant of functional disturbance of the nervous system, appertain to a peculiar type of disease which he proposes to name *nervosism*, "tiré du mot latin *nervosus*, nerveux, pour faciliter l'adjonction souvent necessaire de la double épithète *aigu* ou *chronique*."

Dr. Bouchut tells us that he had often been surprised to hear individuals designated *hypochondriacal* or *hysterical* who were seriously indisposed: men of intelligence who did not exaggerate their sufferings, and who exhibited no signs of disorder in their hypochondria; delicate women who were tormented by the general state of their nervous system, and yet who were not erotic and had no uterine disturbance. It seemed to him, therefore, that a revision of the different groups of the neuroses, which would obviate these practical inconveniences, would be useful to medicine. He has endeavoured to effect this. "Guided," he writes, "by the conscientious and attentive study of the sick, I have been thus led to frame, at the expense of many affections, and especially of *hysteria* and *hypochondriasis*, a general neurosis characterized like those named by the federation of a certain number of nervous disorders of movement, sense, intelligence, and the principal functions." To his own observations Dr. Bouchut has added others furnished to him by his *confrères*, and many which he has culled from the works of Esquirol, Pinel, Chomel, and others; and sundry maladies described by them and still known as certain forms of dyspepsia, gastralgia, mental aberration, general paralysis, delirium, &c., on account of the principal phenomena accompanying the disorders, he finds to be but secondary symptoms of the more complex general disorder of the nervous system, which he has selected and set apart. Again: "the multiform and proteiform nervous contingencies which accompany many nosohæmias or blood changes, and especially chlorosis, which Professor Bouillaud has described with rare precision; certain convalescences, some chronic maladies, and particularly chronic syphilis, which are nearly always exclusively investigated as a consequence of anæmia, without its being considered that this alteration of the blood is as often the result as the cause of the evil:" Dr. Bouchut considers these facts "in a different fashion, because they enter into the category of those that he proposes to study, and he is glad to profit by them."

The curious process of eclecticism here set forth, naturally provokes our curiosity to know somewhat more of the manner in which it was carried into effect. But in this respect we are in a great measure doomed to disappointment, for Dr. Bouchut deals with the subject of his treatise as a well established fact, assuming the truth of his position in the very first sentence, and thenceforth marshalling definition, division, history, causes (predisposing and determining), symptoms, progress, duration, terminations, varieties, complications, and so forth, with charming precision, leaving the reader to discover, by his own ingenuity chiefly, the reasons why such and such results should take their place in systematic medicine.

The question is, however, one of sufficient interest to warrant us in endeavouring to ascertain if there be aught of value, and if so what, in Dr. Bouchut's generalization. In attempting this we shall adopt the order of exposition which he himself has had recourse to.

Dr. Bouchut defines nervosism in the following manner:—

"*Nervosism* is a general neurosis, febrile, or apyretic, characterized by an association, more or less numerous, of variable functional disorders, continued or intermittent, of sensibility, intelligence, movement, and of the chief organic instruments (des principaux appareils organiques).

"These disorders are purely nervous, but they may lead to a belief in the existence of very different organic maladies in the organs of which the function is deranged."—(p. 1.)

It is not often that definitions aid us much in comprehending the nature and character of a disease, consequently, if Dr. Bouchut fails at the onset to give us any clear conception of what he means by nervosism, it is but just to him to imagine that this may possibly arise from his having trusted himself to the treacherous brevity of a definition. It is unfortunate, however, that immediately after the definition we are called upon to admit that the disease so described is found under two forms, one of which, *chronic* nervosism, is said to be familiar to physicians under other names; the other of which, *acute* nervosism, has hitherto been neglected. The acute malady is always accompanied with fever, is infinitely rarer than the chronic one, and rapidly induces the gravest disorders: the chronic malady endures months or years, and when very aggravated may give rise to marasmus and consumption.

Having premised thus much, Dr. Bouchut proceeds to write the history of the disorder, and here we first begin to gain some feeble light as to what he actually aims to teach us.

He tells us that all nervous individuals are not necessarily hysterical or hypochondriacal, as there is too great a tendency to believe from certain writings of Galen, Sydenham, Sprengk, Pomme, &c. The authorities named are a little ancient, certainly, but let this pass. There is another morbid state, we learn, "in which the nervous element plays equally the principal part under a different form, and which can be clearly distinguished by a greater or less number of disorders of intelligence, sensibility, movement, and the chief organic functions, without any appreciable structural alteration of the tissues."—(p. 3.) This is the neurosis that Dr. Bouchut desires to signalize, and which merits to be the subject of special study.

Its existence has been recognised by previous writers, under various names, as for example, *nervous cachexy, marasmus, nervous state, nervous fever*, and *vapours*. It has been confounded with hysteria and hypochondriasis, after the manner of Sydenham, and it constitutes the *nevropathy* of Malcom Fleming; the *hystericisme* of Louyer Villermay; the *névropathie aiguë cérébro-pneumogastrique* of Girard; the *névrospasme* of Brachet; the *névropathie proteiforme* of Cerise; the *nervous diathesis* of many writers, &c. All these terms Dr. Bouchut regards as being objectionable from their tendency to localize the affection, and incommodious from their length; hence he substitutes the solitary word *nervosism.*

Nervosism, however, expresses something more than any of the terms given, taken singly. These are synonyms of different forms of the disorder, rather than of the disorder itself. For this is now seen as an acute affection running a rapid course, and having a serious ending; now it is most justly designated as a diathesis; while anon the proposition of Mead with regard to hypochondriasis aptly fits it: *Non unam sedem habet, sed morbus totius corporis est.* "It is," writes Dr. Bouchut, "the most complex nervous malady that can be produced, and it is not surprising that, in its numerous forms, it has escaped the synthesis of pathologists."—(p. 4.)

Dr. Bouchut conceives that he has fettered this Proteus Morbidus, and that to his questioning unobscure answers have been returned. And truly, if this should prove to be the case, he will have achieved no mean feat; but we fear that the fetters are of sand, and that the oracular responses of the changeable deity are themselves but illusions. Let us glance at the disease as Dr. Bouchut depicts it:—

"Confounded even now with hysteria or with hypochondriasis by all those who systematically term thus the nervous ills observed in woman, man, and even the infant, often with chronic gastritis, and with gastralgia, with epilepsy, mental alienation, dyspepsia, organic maladies of the brain, of the spinal marrow, of the heart, &c., &c.; nevertheless it is very different from these maladies. It approaches to and removes afar off from at one and the same time the neuroses and certain organic diseases. We encounter there paralysis, contractions, tonic and clonic convulsions, tremblings, spasms, fainting-fits, syncope, neuralgias, visceralgias, disorders of the intelligence and the organs of sense, such as sensorial illusions or hallucinations, simple or hectic fever; and it is but the mode of apparition, development, and succession of these morbid phenomena which reveals to us their true nature. In this respect they merit the attention of physicians, who will doubtless recognise a very common malady, which is often a source of the greatest embarrassment to them in practice."—(p. 5.)

We have every respect for Dr. Bouchut, but does not this last sentence give to *nervosism* the character of being a word, and nothing more, provoking us to exclaim, with M. Jourdain, " Par ma foi, il y a plus de quarante ans que je dis de la prose, sans que j'en susse rien; et je vous suis le plus obligé du monde de m'avoir appris cela?"

Among ancient writers no one appears, to Dr. Bouchut, to have described clearly and precisely *nervosism*, except Hippocrates. He touched upon the subject incidentally, but unfortunately this earliest recognition of the affection was " étouffées" by the chimerical hypotheses of Galen upon hypochondriasis. Dr. Bouchut recognises *nervosism* in the description which Hippocrates gives of the nervous disorders accompanying inanition, seminal emissions, and gastralgia. Notwithstanding, however, Dr. Bouchut's dictum, that *nervosism* was " seen imperfectly and summarily described by Hippocrates," we cannot say, with Géronte, in *Le Médecin malgré lui*, " Puisque Hippocrate le dit, il le faut faire;" neither can we admit that since Hippocrates' time the nervous ailments he described under the circumstances named, have been " confounded with different morbid, and, according to some symptoms, nearly similar states."

"His numerous examples," writes Dr. Bouchut, "have always been classed in a vicious fashion, and designated by inappropriate names in relation with certain reigning ideas. It would be a curious study to make, that of the influence of words upon the things that they represent, and of the evil effects of a bad denomination upon the progress of science. There is not a physician who has not observed examples of a general nervous malady, entirely distinct from hypochondriasis and from hysteria; but for want of a word, every one confounds the first of these morbid states with the two following, or still more wrongly, with gastritis, gastralgia, chlorosis, anæmia, diseases of the heart, or spinal marrow, according to the ideas in vogue at the moment."—(p. 10.)

We shall presently see how Dr. Bouchut's suggestive remarks on vicious designations and their influence, may usefully be brought to bear in testing the merits of his present work.

Robert Whytt was, according to Dr. Bouchut, the first person who attempted to separate nervousness from hysteria and hypochondriasis. Whytt wrote:—

" Persons liable to perturbations of the nerves, some of which deserve the name of *nervous* much better than others, may be distinguished into *three classes*."

And a little beyond :—

"The complaints of the first of the above classes may be called *simply nervous;* those of the second, in compliance with custom, may be said to be *hysteric;* and those of the third *hypochondriac.*"—(p. 14.)

Now Dr. Bouchut asserts that "the efforts of Robert Whytt have unhappily remained unfruitful, in this sense at least that they have not served as a rule to nosography." The reservation is just, for we presume that Dr. Bouchut would hardly state, that the division adopted by Whytt is not the one practically in use among physicians, as well in France as in England. And whereas *hysteria* and *hypochondriasis* are the only two of the three classes into which Whytt divides the nervous perturbations to which he refers, that present any constant positive characteristics, they alone have been promoted to a position in our nosographies. Why the class specially called *nervous* should have been excluded, and whether it should now receive a fitting place, will be better seen when we have proceeded somewhat further with our examination of Dr. Bouchut's treatise.

The *predisposing* and *occasional causes* of nervousness are next treated by Dr. Bouchut at considerable length. Age, sex, menstruation, pregnancy, suckling, uterine maladies, &c., the nervous temperament, heritage, original or acquired feebleness, education, the passions, watchings, excess of work, venereal excesses, convalescence from acute and chronic maladies, anæmia, hydrohæmia, and chlorosis, all receive due notice under these heads. It is not needful that we should track Dr. Bouchut's remarks over this ground, but we may cull a few sentences here and there which will serve to throw additional light upon his opinions. He tells us that "there are few acute and especially chronic maladies, in which the nervous state does not primarily or secondarily play an important part." We also learn that—"the *nervous temperament* which predisposes the organism to the different known disorders of the organs of innervation, favours more than any other cause the development of the morbid state of which he speaks. It seems in truth that the multiplicity of morbid phenomena of the nervous state are more in relation with the general constitution of the subject than the appearance of convulsions or of essential paralyses, for example."—(p. 25.)

Further we are told that—

"The majority of chronic diseases, and especially those of the intestine and of the stomach, sometimes also of the uterus, can give birth to acute or chronic nervosism. Indeed, although this state may be *primitive*, it is much oftener *secondary*, and it depends very frequently upon a material somatic lesion, of which the sympathetic or reflex action is manifested by vague disorders, mobile and multiple, in the nervous system."—(p. 31.)

Again, Dr. Bouchut sums up his remarks upon the occasional and predisposing causes of nervosism, by saying :—

"Finally : although the numerous causes of acute or chronic rheumatism are very varied in their nature and appearance, very different the one from the other, since we find natural or acquired feebleness by the side of the predisposing influence of age, of heritage, of chagrins, of passions of every kind, of convalescence, of hæmorrhage, or a chronic malady, they are all held together more or less by a common bond, which is the diminution of the *quantity* or of the *quality* of the total mass of the blood, and the decrease of one or other of its elements, particularly of its globules. There is nearly always at the bottom of nervosism primitively or secondarily, a modification more or less considerable of the blood, and of the organic crasis."—(p. 32.)

These quotations, although they deal with very familiar matters, do not, however, yet enable us to form any very definite ideas of what *nervosism* consists in. We have now, however, arrived at the point where Dr. Bouchut introduces the symptoms of the disorder, and may consequently hope to see our way more clearly to the notion he wishes to convey.

And first of the symptoms of *acute nervosism.*

This, it appears, is a very rare disease. It is ushered in by malaise, accompanied by feebleness, loss of appetite, and disgust of food, sometimes also by ptyalism, nausea and aqueous vomitings, obstinate constipation, and general irritability with fever. The patient's strength is exhausted, and he is compelled to keep his bed. Some cannot raise the head from the pillow without fear of faintness or syncope. Odours, noises, and light are supported with difficulty. The senses, becoming very excitable and morbidly sensitive, occasion suffering when exercised, or give rise to numerous sensorial illusions, especially at an advanced period of the disease. Then wasting and alteration of the lineaments become conspicuous, the tongue is blanched, the vomitings continue, and the constipation persists, as well as the febrile state characterized by acceleration of the pulse and heat of the skin. Next, very grave neuralgic and cerebral accidents supervene; general or partial pains in the head or in the limbs, delirium at first transitory, then continued, enfeeblement of the organs of the senses, singular hallucinations, drowsiness, coma, rigidity of the muscles, convulsions, and in the end death after two or three months of unheard-of sufferings. In fatal cases the most scrupulous examination fails to discover any appreciable structural alteration in the principal organs.

In this general description of acute nervosism we have followed Dr. Bouchut's account almost textually. But this will be further elucidated by copying one of the cases which he quotes in illustration of the affection. We take the first recorded :—

"A lady, about forty years of age, mother of a family, wife of a member of the Academy of Sciences, was, at the climacteric period, enfeebled by profuse uterine hæmorrhages.

"In the winter of 1846, as the sequel of slight bronchitis which necessitated repose and regimen, this lady, who was extremely impressionable, did not recover her appetite, and continued to be feverish. An extreme irritability, bizarre ideas, very acute sensitiveness of the eyes to the light of day, hyperæsthesia of the ears and of the skin upon the limbs, neuralgic pains of the head, sleeplessness, nocturnal alarms, and considerable muscular debility, caused by amyosthenia, were observed.

"The patient wasted visibly, and she could not take anything, being disgusted even with drinks. She was fatigued by an abundant and mucous ptyalism. There was neither vomiting nor alvine evacuation. The skin was warm, and the pulse, small and frequent, beat 120 times in the minute.

"This state of things persisted one month. It was now December, and although *bouillon* was not digested, I ordered a cutlet, after an immersion in water at fifteen degrees, and a forced promenade of ten minutes duration in the street at a time of terrible snow. I prescribed also a daily slight dose of sub-carbonate of iron.

"This medication succeeded very well, and every day was signalled by a new amelioration in the increase of strength and of embonpoint, and in the diminution of nervous excitability.

"Nevertheless, four months of hydro-therapeutic treatment, of the use of ferruginous preparations, and lastly a sojourn in the *campagne des Moulineux*, near Paris, were required in order completely to restore the patient."—(pp. 54, 55.)

Dr. Bouchut next describes *seriatim*, and at length, the different symptoms observed during the progress of the so-called acute nervosism; but these do not aid us much to a clearer knowledge of the affection. He then proceeds to discuss the chronic form of the disease.

This, we are told, is infinitely more common than the acute one, and "is found at every step in civil practice, there being few women of the world who do not present some symptoms of it, without being on this account completely ill."—(p. 92.) Dr. Bouchut proceeds:—

"It presents infinite degrees like other diseases, and just in the same manner that we may observe many symptoms of scroful*ism*, lymphat*ism*, and podagr*ism*, &c., &c., without having either scrofula or gout as a well-defined malady, we observe among a great number of persons habitual nervous troubles which announce the diathesis, without still specifying the chronic nervous state which may be developed, somewhat later, with great intensity. Nothing is so common as the first degree of the malady, a true exaggeration of the nervous temperament; but when this previous disposition is aggravated under the influence of the moral or physical causes of which I have spoken, the nervous phenomena are multiplied, and become more violent as well as generalized (*se generalisant*), the functional disorders, at first easily governed on account of their slight importance, become intolerable, and the whole of the perturbed organization becomes the theatre of numerous, varied, and often very grave accidents.

"The manifestations of the diathesis are multiplied to infinity, and the functions of the general or special sensibility, of intelligence, movement, of respiration, circulation, digestion, of the secretions singly or simultaneously disordered, give rise to a great number of symptoms highly characteristic of the disease."—(p. 92.)

The very various psychical and physical symptoms thus referred to are too familiarly known under the designation *nervous*, in its widest sense, to require recapitulation. It is needful, however, to note that they may occur *primarily*, to wit, "distinct from all natural visceral complication, or *secondarily*, that is to say, provoked by an acute or chronic, a tubercular, epithelial, or cancerous *nosorganie*, the symptoms being very nearly similar, except the presence of some phenomena intimately bound to the anterior morbid state."—(p. 93.)

In a malady manifested by so many and such varied symptoms, it may be surmised that the simple division into an acute and chronic, and again into a primitive or secondary affection, will hardly suffice to give a complete idea of its character, and the different forms it may assume, determined by the affections with which it is linked, or by the predominance of certain symptoms.

Thus in the course of pregnancy and of convalescence, under the influence of chlorosis and gastro-intestinal maladies, nervosism exhibits interesting peculiarities which cannot be neglected. The same is also true when certain nervous disorders predominate; and Dr. Bouchut suggests, interrogatively, the propriety of adopting the following subdivisions of the affection:—

1. *Cerebral nervosism*, characterized by vertigo, giddiness, severe pain in the head, partial or general paralysis, sensorial illusions and hallucinations. This form readily simulates apoplexy and diseases of the brain.

2. *Spinal nervosism*, accompanied by disorder of the sensibility and motility of the pelvic members, simulating disease of the spine.

3. *Cardiac nervosism*, determined by the constant presence of palpitations or faintings, and very frequently regarded as an organic affection of the heart.

4. *Laryngeal nervosism*, in which considerable cough or aphonia is observed, and which is sometimes considered as the commencement of phthisis.

5. *Gastric nervosism*, chiefly indicated by dyspepsia, heartburn, moderate or uncontrollable vomiting, &c., long considered as a form of gastritis, and treated as such.

6. *Uterine nervosism,* signalized by lumbar and inguinal pains, by sensation of weight in the perinæum, and by leucorrhœa, independent of any structural lesion, but which is apt to be regarded as chronic metritis.

7. *Cutaneous nervosism,* in which the predominant phenomenon observed is hyperæsthesia of the skin.

8. *Spasmodic nervosism,* giving rise to spasms in a great number of tissues and organs.

9. *Paralytic nervosism,* distinguished by general or partial abolition of movement and of sensibility in the muscles of the members, or in the sensory organs.

10. *Painful nervosism — nervosisme douloureux* —including *temporal, maxillary, occipital, auricular, intercostal neuralgia,* &c., a variety corresponding with the *neuralgic* affections of most medical writers.

Dr. Bouchut adds that the propriety of accepting these subdivisions is doubtful in the actual state of science, and that to resolve the question further studies will be necessary. But the suggestions are of interest from the light which they throw upon the signification which Dr. Bouchut would have us attach to the word *nervosism.*

We pass over the chapters which treat of the complications and pathological anatomy of the affection, as these aid us but little in acquiring a knowledge of it, and we come next to its diagnosis.

Many diseases, Dr. Bouchut tells us, may be confounded with nervosism in its acute or chronic state, either by their primary symptoms or those which are occasioned secondarily by a complication. Among the diseases that may thus be misinterpreted are, *hysteria, hypochondriasis, monomania, dementia, dyspepsia, gastralgia, constitutional syphilis,* &c. We shall confine ourselves solely to the discrimination of the three affections, hysteria, hypochondriasis, and insanity, from nervosism.

Hysteria and chronic nervosism are two diseases, Dr. Bouchut states, equally common among females, and which have several symptoms in common. Nevertheless, hysteria is an essentially convulsive neurosis, in which tears, spasms, convulsions, and loss of knowledge of a peculiar character, play the principal part; whilst in nervosism faintings are exceedingly rare, as well as convulsive attacks resembling eclampsia, and which are not always accompanied by unconsciousness. Hysteria gives rise to a sensation as of a ball rising in the throat, a phenomenon unknown in nervosism. Hysteria occurs in apyretic paroxysms at greater or less intervals; nervosism as a persistent disorder, sometimes with fever irregularly intermittent, sometimes without fever when the disease is not very intense. Hysteria never suspends nutrition, whilst nervosism may arrest it and occasion marasmus. Both affections, however, manifest in common certain disturbances of intelligence, motion, general or special sensibility and of the secretory organs. Delirium, agitating dreams, hallucinations, sensorial illusions, paralysis of the muscles, or of the organs of sense, hyperæsthesia, or anæsthesia of the skin, intense neuralgia, superficial or deep-seated, clear and abundant urine, &c., are observed in both diseases, according to their form and the intensity of the morbid condition.

*Hypochondriasis* is particularly characterized by the constant pre-occupation of the patient with real or imaginary sufferings, and never exists in an acute state. The melancholy and sadness, the fear of death from indeterminate sufferings, whether profound or not, which are observed in this disease are never witnessed in the same degree in chronic nervosism, and the neuralgic pains which occur in the latter affection are of a severer character than those which happen in the former. Delirium, sensorial illusions, very rarely supervene in hypochondriasis, and never convulsions, muscular rigidity, or paralysis; never swoonings, nor fever, nor marasmus; but both affections have alike dyspepsia, constipation, shortness of breath, palpitations of the heart and arteries, and general loss of strength. "The development by an acute state followed by a chronic condition, the presence of fever and the intensity of the pains and of the principal nervous phenomena, are the most important differential characters of the two neuroses."

We have adhered almost literally to Dr. Bouchut's account of the diagnosis of hysteria and hypochondriasis; we must, however, give a still more faithful rendering of his remarks on the diagnosis of insanity. He writes:—

"Many forms of insanity, monomania, for example, may be easily confounded with acute or chronic nervosism, because they reproduce some of its most important symptoms. Without speaking of acute febrile delirium which every one now separates from mental alienation, there is in certain diseases a partial monomaniacal delirium, characterized among some patients by a tendency to suicide, among others by hallucinations or sensorial illusions of the touch, sight, taste, hearing, or smell, followed by unreasonable acts which are sometimes hardly to be distinguished from insanity. It is principally by their rapid progress and short duration that the nature of the accidents of nervosism can be recognised. In reality, the monomaniacal madman has all the appearances of health, and is but rarely feverish; his digestion is generally good, and he does not manifest either disorder of sensibility or of movement. All the indisposition is confined to the disorder of the faculties and understanding; it has a chronic progress, and the remainder of the organization takes no part in it. In acute or chronic nervosism, on the contrary, the intellectual disturbance is entirely secondary, transitory and consecutive to grave functional disorders of all the organs; it is indeed an epiphenomenon in the middle of a morbid state already well defined."—(pp. 269-70.)

Dr. Bouchut treats duly of the prognosis, nature, and treatment of nervosism in the three terminal chapters of his work, but we gain little additional light from them upon the nature of the supposed affection. One remark, however, touching the connection of nervosism with blood-changes, may be quoted with advantage from the penultimate chapter:—

"Nervosism is not necessarily accompanied by alteration of the blood, and when this exists, it is not always similar, since chlorotic, gouty, syphilitic, or herpectic nosohæmia, &c., may be the point of departure. Moreover, anæmia, which is so often regarded as the absolute cause of the nervous state, or nervosism, has not this importance, because it is not always present at the beginning of the symptoms, and in most cases it is a secondary element, or an effect of the principal malady."—(p. 207.)

We think now that we have written sufficient to put our readers in a position to form a tolerably definite judgment of the signification which should be attached to the term *Nervosism*, and of the value of the generalization which that word expresses. It is evident that Dr. Bouchut includes under the term *all* the so-called nervous symptoms which are not included under the expressions hysteria and hypochondriasis in their most restricted sense. But it is a little difficult to conceive why these affections should have been excluded from the generalization. Dr. Bouchut, we presume, uses the term *nervous* much in the same way as Whytt himself, who after remarking that all diseases may in some sense be termed nervous, writes:—"However those disorders may peculiarly deserve the name of NERVOUS, which on account of an unusual delicacy, or unnatural state of the nerves, are produced by causes which, in people of sound constitution, would either have no such effects, or at least in a much less degree."* Dr. Cullen has a comment upon this observation of Whytt's which is worthy of quotation. He writes:—

"Dr. Whytt, who has treated this subject (the General Pathology of the Nervous System) *ex professo*, observes the difficulty there is in limiting the subject, as all diseases may, in a certain sense, be called affections of the nerves. Every preternatural state, either of sense or motion, depends upon the nervous system, so that the nerves are more or less concerned in every disease; and this title might consequently

* *Observations on the Nature, Causes, and Cure of those Disorders which are commonly called Nervous, Hypochondriac, or Hysteric.* By Robert Whytt, M.D. Collected Works. Edin. 1768, p. 529.

comprehend the whole of diseases. But some diseases are more strictly termed nervous, and some limits have been tacitly assigned or conceived in the minds of physicians. Those diseases, the symptoms of which appear only or chiefly in the nervous system itself, which appear purely in the functions of sense and motion, and in which the sanguiferous system is not necessarily or is only occasionally affected, such may be called more strictly nervous diseases; so that the whole of the febrile diseases, however much they may be said to consist in affections of the nervous system, are, by this limitation, excluded."

Now this is really the sense in which the term *Nervous* is still used by physicians, consequently hysteria and hypochondriasis are rightly included under the general head of nervous diseases. Hence admitting *nervosism* as a legitimate generalization, it would be more consistent with the spirit of the generalization to add the two affections to nervosism, regarding them as forms of that disease, and designating them *hysterical* and *hypochondriacal nervosism.*

"Of two things one," writes Dr. Bouchut, "either hysteria and hypochondriasis are different neuroses, or they are similar. If they be similar, which I do not believe, nervosism need not be studied apart; but if on the contrary, the two diseases are distinct, it is needful to admit that there is a particular form of neurosis, a species of nervous diathesis or *nervosism* which should not be confounded with them."—(p. 290.)

Suppose, however, that hysteria and hypochondriasis are two different *forms* of manifestation of one and the same diathesis—using that word in the sense ordinarily received? Dr. Bouchut's dilemma entirely overlooks this supposition, which, singular to say, is the one most generally indulged in, and the one adopted by Whytt himself, to whom Dr. Bouchut refers with such great respect. Whytt tells us that, in treating of nervous disorders, he shall confine himself—

"Chiefly to those complaints which proceed, in a great measure, from a weak or unnatural constitution of the nerves; and of this kind, I presume, are most of those symptoms which physicians have commonly distinguished by the names of *flatulent*, *spasmodic*, *hypochondriac*, or *hysteric.*

"As the sagacious Sydenham has justly observed, that the shapes of protæus, or the colours of the chamæleon, are not more numerous and inconstant than the variations of the hypochondriac and hysteric disease; so those morbid symptoms which have been commonly called *nervous*, are so many, so various, and so irregular, that it would be extremely hard either rightly to describe or fully to enumerate them. They imitate the symptoms of almost all other diseases; and, indeed, there are few chronic distempers with which they are not more or less blended or intermixed. Hence it is that the late Dr. Mead says of the hypochondriac affection, *Non unam sedem habet, morbus totius corporis est.*"*

Then Dr. Whytt proceeds to enumerate the chief of the many varied symptoms which characterize nervous disorders, and he writes :—

"Patients after having been long afflicted with many of these symptoms (for all of them never happen to any one person), sometimes fall into melancholy, madness, the black jaundice, a dropsy, tympany, *phthisis pulmonalis*, palsy, apoplexy, or some other fatal distemper. Some patients who are liable to the above complaints, some of which deserve the name of *nervous* much better than others, may be distinguished into three classes.

"1. Such as, though usually in good health, are yet, on account of an uncommon delicacy of their nervous system, apt to be often affected with violent tremors, palpitations, faintings, and convulsive fits, from fear, grief, surprise, or other passions; and from whatever greatly irritates or disagreeably affects any of the more sensible parts of the body.

"2. Such as, *besides being liable to the above disorders from the same causes* [suffer from *hysterical* symptoms properly so called].

"3. Such as, from a less delicate feeling or mobility of their nervous system in general, are scarce ever affected with violent palpitations, faintings, or convulsive motions, from fear, grief, surprise, or other passions; but, on account of a disordered state of the nerves of the stomach and bowels, are seldom free from complaints of indigestion, belching, flatulence, want of appetite, or too great craving, costiveness, or looseness, flushings, giddiness, oppression, or faintness about the *præcordia*, low spirits, disagreeable thoughts, watching, or disturbed sleep, &c.

"The complaints of the first class may be called *simply nervous;* those of the second, in compliance with custom, may be said to be *hysteric;* and those of the third, *hypochondriac.*"†

And, again, he writes of *hysteria* and *hypochondriasis :*—

"Whether these two distempers be considered as the same or distinct, since the symptoms of both are so much akin, we shall consider them under the general character of *nervous.*"‡

Here then we have Whytt regarding hysteria and hypochondriasis as different *forms* of manifestation of a general nervous state, while to the indications of that state, *not* of an hysteric or hypochondriac character, he proposes, for convenience sake, to speak of as *nervous par excellence.* This mode of viewing these affections has been retained to the present time, and the intimate mode in which the three different forms are linked the one to the other, explains how it has happened that the domains of hysteria and hypochondriasis have been so undefined,

* *Op. cit.*, p. 530. † *Op. cit.*, pp. 532–33.
‡ *Op. cit.*, p. 534.

and also how individuals who have given their special attention to the one or other form of disease, have often contrived to include in its manifestations many symptoms not peculiar to it. There can be no doubt, however, of the propriety of the substantive terms *hysteria* and *hypochondriasis*. Typical forms of these affections are by no means of uncommon occurrence. But do the symptoms to which Whytt restricted the term nervous ever occur in so definite a form, that we may with fitness give to the whole of them a substantive name as Dr. Bouchut proposes?

The word *nervosism* which he suggests for such a purpose, nearly coincides in meaning with that of our popular substantive *nervousness*, and would be most accurately rendered by that word. But the necessary vagueness of use of the latter word would be exceeded by that of the former one, because it certainly includes a greater variety of symptoms. If we except, for the moment, Dr. Bouchut's subdivision *acute nervosism*, we cannot lay hold of a single group of symptoms which he details under the head *chronic nervosism* which is not better described by the simple and accustomed epithet nervous. This adjective expresses a general fact, and the word, on its face, neither implies nor conveys an idea of more accurate knowledge of the phenomena that it refers to than we possess. But if we put in place of it a substantive, as for example, *nervosism*, we at once imply a well-defined affection, which is not warranted by the symptoms. We should, indeed, convey the appearance of knowledge, when the reality is far from us.

It may be said, however, that in the *acute nervosism* of Dr. Bouchut, we find a clearly marked acute affection. We have quoted the first instance cited by Dr. Bouchut, and leave it to our readers to judge of its value as supporting a proposition for the recognition of a new and "very rare" form of disease.

Dr. Bouchut's observations on the diagnosis of *nervosism* partake too clearly of the method of selection which has governed his generalization to be of any value. Does terming the transitory insanity, which is occasionally observed in persons of a highly nervous diathesis, become any the less insanity by being termed *nervosism*? Does observation teach us that hysteria is trenchantly defined by the hysterical paroxysm; or that the absence, under given circumstances, of fever, wasting, and nervous accidents commonly so called, is essential to constitute a case of hypochondriasis? Dr. Bouchut certainly deserves no small credit for the ingenuity with which he has, as he phrases it, "at the expense of many nervous affections," constituted a new form of disease out of old material; but the work is not the less a work of ingenuity rather than of observation. We demur to any such method of improving our nosological arrangements unless it can be clearly shown that it might aid as a stepping-stone to further research. Dr. Bouchut's work cannot be looked upon in such a light. To accept his generalization would be to adopt an expression calculated to cast a false light upon a class of phenomena, than which we know nothing that presents so interesting a field for, and which so well repays accurate observation and research.

It may be thought that we are giving more attention to Dr. Bouchut's work than is necessary, but truly to some persons newly-invented technical words have an irresistible charm, as if they possessed an occult property. Dr. Bouchut has well suggested, as we have already seen, the harmful influence which words exert at times upon science, and we wish, as far as in us lies, to guard his new word *nervosism,* from having like injurious results. We do not think that our neighbours require the word, and for those among ourselves who are not content to clothe their ideas concerning the phenomena of which we have been writing, in language which would neither fall short of nor exceed the knowledge they possess of them, the time-honoured word *nervousness* ought to suffice.

---

